# Antimicrobial prescription KAP among physicians in primary care institutions in Southwest China

**DOI:** 10.1371/journal.pone.0335484

**Published:** 2025-11-13

**Authors:** Yun Lu, Junli Yang, Yuxing Yan, Yue Chang

**Affiliations:** 1 School of Public Health, the key Laboratory of Environmental Pollution Monitoring and Disease Control, Ministry of Education, Guizhou Medical University, Guiyang, Guizhou Province, People’s Republic of China; 2 School of Medicine and Health Management, Guizhou Medical University, Guiyang, Guizhou Province, People’s Republic of China; 3 Center of Medicine Economics and Management Research, Guizhou Medical University, Guiyang, Guizhou Province, People’s Republic of China; Al-Ahliyya Amman University, JORDAN

## Abstract

**Objectives:**

The current status of antimicrobial prescription knowledge, attitudes, and practices (KAP) landscape regarding antimicrobial prescriptions among the physicians in southwestern China was evaluated in this study. The objective of this study was to pinpoint the weak areas in the physicians’ antimicrobial prescription KAP and provide targeted recommendations.

**Methods:**

In this cross-sectional study, a multi-stage sampling method was adopted to select 192 primary care institutions from 9 prefecture-level administrative divisions in southwest China, and questionnaires were distributed to the physicians within these institutions. The KAP score of antimicrobial prescription among the physicians was calculated. Variables on the demographic characteristics of the physicians were also collected. For comparisons between groups on single-factor variables, t-tests or one-way analysis of variance, while multiple linear regression was utilized to further explore the influencing factors.

**Results:**

A total of 518 physicians were included in this study. The physicians’ average scores for antimicrobial prescription KAP were 3.98 ± 1.85 (<50%, poor level), 41.97 ± 4.59 (50–79%, average level), and 40.01 ± 5.63(>80%, good level), respectively. Multivariate analysis revealed that work duration significantly influenced physicians’ antimicrobial prescription knowledge and attitude levels, whereas sex was the primary factor affecting their antimicrobial prescription attitude and practices levels (*P* < 0.05).

**Conclusion:**

Among the physicians in southwest China, the overall score for antimicrobial prescription knowledge was relatively low, the scores for prescription attitudes and practices were generally average and high. These results provide valuable insights for relevant departments and institutions regarding physicians’ antimicrobial prescription KAP in primary care institutions, thereby guiding the optimization of policies and the development of targeted interventions.

## 1. Introduction

Antimicrobial resistance has become one of the public health challenges worldwide. Current global surveillance data reveal that antimicrobial resistant infections already account for 700,000 annual mortality cases. It is estimate that antimicrobial resistance could lead to approximately 10 million fatalities worldwide by 2050 [1].

Overuse and misuse of antimicrobial agents by medical professionals is one of the primary drivers of antimicrobial resistance [2]. Therefore, it is crucial for medical personnels to comprehend the appropriate use of antimicrobial agents and the mechanisms underlying antimicrobial resistance, as this understanding can contribute to mitigating the issue of antimicrobial resistance [2–4]. The research on knowledge, attitude, and practice (KAP) concerning antimicrobial prescriptions aims to provide an in-depth examination of this critical issue. Studies in developing regions, such as Asia, indicate that while 79.5% of physicians possess a good level of antimicrobial agents knowledge, only 27.8% demonstrate appropriate prescriptions practices [5–6].

On the other hand, previous systematic review study have demonstrated antimicrobial prescription KAP have primarily focused on the physicians in tertiary hospitals and specialized hospitals, few surveys have addressed physicians in primary care institutions [7]. As a developing country, China’s southwestern region is characterized by relatively scarce resources, and there is a paucity of studies examining the prescription KAP of physicians. In China, primary care institutions handle 53.23% of the national outpatient volume [8]. Physicians in these institutions, as key stakeholders in antimicrobial prescriptions and dispensation, the physicians play a pivotal role in controlling the inappropriate use and resistance of antimicrobial agents [9].

This study investigates the prescription KAP of physicians in primary care institutions in southwest of China, aiming to identify weak links and related influencing factors in their antimicrobial prescription-related knowledge and practices. The findings will provide a critical reference for formulating and optimizing future policies, education and training programs, ultimately contributing to the optimization of antimicrobial agents use and control of antimicrobial resistance in primary care institutions.

## 2. Methods

### Setting, design, and population sampling

**Fig 1:**
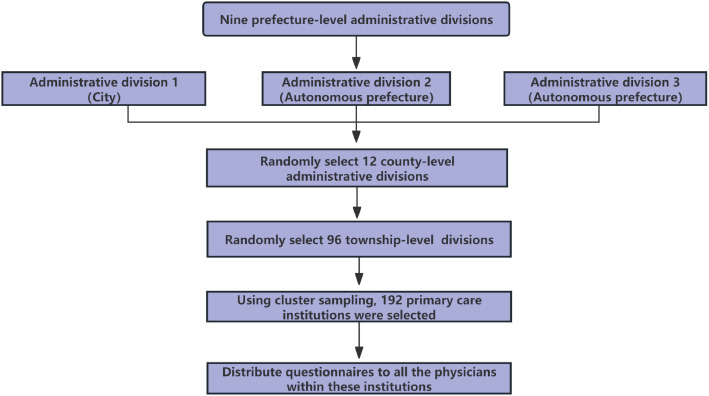
Sampling flowchart.

This cross-sectional study was conducted among physicians working in public primary care institutions across southwest of China, with physicians as the survey respondents. The survey was carried out from December 23, 2024, to January 18, 2025. China’s administrative structure comprises three levels: prefecture-level, county-level, and township-level divisions. A multi-stage sampling approach was adopted to survey the physicians in southwest of China. In the first stage of this study, prefecture-level administrative divisions were selected as sampling units. Three prefecture-level administrative divisions were chosen from the nine prefecture-level administrative divisions in southwest of China using simple random sampling (Lottery method). In the second stage, four county-level administrative divisions were randomly selected (Random number table method) from each of the three chosen prefecture-level administrative divisions, totaling 12 county-level divisions. In the third stage, eight township-level administrative divisions were selected from each of the 12 county-level administrative divisions (Random number table method), resulting in a total of 96 township-level divisions. In the fourth stage, cluster sampling was used to select two primary care institutions from each of the 96 township-level administrative divisions, leading to a total of 192 primary care institutions. A survey was conducted among the physicians from these institutions (Fig 1).

### Sample size

The sample size was calculated using the formula to cross-sectional studies. According to the survey results conducted by He Jin et al [10], the awareness rate of antimicrobial agents among Chinese physicians is approximately 50.8%. This value was then incorporated into the formula for the calculation.


$$n=\frac{Z_{1-\alpha /2}^{2}\times P\times (1-P)}{\delta^{2}}$$

The significance level was set at *α* = 0.05, with *Z*(1-*α*/2)=1.96, *δ* = 0.1*P*, *P* = 50.8%, and the permissible error was 0.0508. Based on these parameters, the calculated sample size was approximately *n* ≈ 373. To account for a potential 10% loss to follow-up, the sample size was adjusted to *n* = 411. In order to further enhance the reliability of the data, we increased the sample size to no fewer than 500 physicians.

### Participants

The inclusion criteria for physicians were as follows:

① Physicians who had worked in primary care institutions for at least one year;
② The number of outpatient visits per 10-day period should not be fewer than 100;
③ Participated in this study on a voluntary basis.

Exclusion criteria for physicians:

① Refresher physicians (This refers to the group of newly recruited physicians who need to regularly participate in further education and training to update their professional knowledge and maintain their clinical skills [11]).

Physicians meeting the inclusion and exclusion criteria in these selected institutions were surveyed, a total of 518 physicians were included in the study.

### Questionnaire

The questionnaire was developed based on the 2015 edition of China’s Basic Principles for the Clinical Application of Antimicrobial agents (S1 Appendix), the United States Centers for Disease Control and Prevention (CDC) Guidelines for use of antibiotics [12], and the WHO questionnaire for antibiotic resistance: multi-country public awareness survey [13]. First, it was administered in Chinese, and its reliability and validity were confirmed to meet established standards. Internal consistency reliability was assessed using Cronbach’s alpha, yielding a coefficient of 0.70, which is considered acceptable. Construct validity was evaluated, with the KMO value reaching 0.81 (*P* < 0.05), exceeding the threshold of 0.7, as supported by Bartlett’s test results. Second, the questionnaire was piloted with 20 physicians from the other primary care institutions who met the inclusion and exclusion criteria, and these physicians were subsequently excluded from the analysis. Third, the answers to the questionnaire in this study were strictly formulated based on clinical guidelines rather than subjectively set, ensuring the accuracy of the responses. On the other hand, the questionnaire underwent a pre-survey. Only after physicians confirmed that it could truly reflect the KAP level of antimicrobial agents in primary-level regions was the formal survey initiated, ensuring the validity of the questionnaire.

A 43-item questionnaire was developed and structured into four distinct sections. Section one focused on basic demographic information, comprising 11 questions related to participants’ gender, age, and marital status. Section two addressed the prescription knowledge of antimicrobial agents, consisting of 11 questions. This section covered topics such as the antimicrobial agents spectrum of penicillins, contraindications for fluoroquinolones, adverse reactions associated with tetracyclines, and awareness of antimicrobial resistance. Section three examined attitudes toward antimicrobial prescription practices, including 11 questions that explored whether the physicians would conduct blood or bacteriological tests before prescribing antimicrobial agents, prescribe antimicrobial agents as prophylactic medication for upper respiratory tract infections, or and whether to reduce the dose of antimicrobial agents because of their side effects. The final section, section four, evaluated antimicrobial prescription practices, encompassing 10 questions. These questions assessed scenarios such as prescribing antimicrobial agents for patients with fever of unknown origin or in response to patient expectations. In China, the use of aminoglycoside antimicrobial agents is relatively common in primary care institutions. Furthermore, intravenous administration of antimicrobial agents is also more prevalent in primary care institutions. Accordingly, these two questions have been selected to assess the knowledge, attitudes, and practices (KAP) of physicians regarding antimicrobial prescription [14–15].

The questionnaire was structured into three dimensions: prescription KAP, with a total score of 116. The prescription knowledge dimension had a maximum score of 11, with the minimum possible score being 0. The prescription attitude dimension had a maximum score of 55, with the lowest possible score being 11. The prescription practice dimension had a maximum score of 50, with the lowest possible score being 10. Higher scores indicated better antimicrobial prescription knowledge, more positive prescription attitudes, and better prescription practices among the study subjects. For the prescription knowledge dimension questions, single-choice questions were scored as 1 point for correct answers and 0 points for incorrect answers. Multiple-choice questions were awarded 1 point only if all correct answers were selected. Partial or incorrect answers received 0 points. Questions related to the physicians’ prescription attitudes and practices were scored on a five-point Likert scale, where 1 represented the least appropriate response and 5 represented the most appropriate response. Some questions were reverse-scored to account for unfavorable responses. For instance, if the statement of some questions was positive, a response of “strongly agree” would earn 5 points. However, if the statement in the question was negative, a response of “strongly agree” would only earn 1 point. Using the modified Bloom’s cut-off point, the KAP scores of physicians’ prescriptions for antimicrobial agents were divided into three levels: good (scores ranging from 80% to 100%), average (scores ranging from 50% to 79%), and poor (scores below 50%) [16].

### Data collection

Each participant was permitted to complete the questionnaire only once in our study. And data collection was conducted via both the Questionnaire Star electronic platform (www.wjx.cn) and paper-based questionnaires administered offline. Researchers explained the background and objectives of the survey to the physicians before distributing the questionnaire. Participants were given 30 minutes to complete the questionnaire and had the option to withdraw from the survey at any time.

### Data analysis

SPSS 27.0 software was utilized for data analysis. The data set conformed to a normal distribution, and measurement data were presented as mean ± standard deviation (Mean ± SD) in descriptive analyses. Count data were summarized using frequencies and percentages. For comparisons between groups on single-factor variables, t-tests or one-way analysis of variance (ANOVA). In accordance with the standards in the reference, variables with *P* < 0.2 from the uni-variate analysis were included in the multivariate analysis [17–18]. This is a common practice in the field of public health, as it allows for the incorporation of more variables into the multivariate analysis. Multiple linear regression models were constructed to identify factors influencing antimicrobial prescription KAP among the physicians in southwest of China. The rationale for conducting further multivariate analysis lies in the fact that univariate analysis only evaluates the effect of a single factor (independent variable) on the outcome (dependent variable), without accounting for potential confounding variables or possible interactions among variables. To confirm the robustness of the findings, multivariate analysis is commonly performed [19–20].

### Ethics approval and consent to participate

All respondents provided written informed consent before participating in the study. Participation was strictly voluntary and anonymous, with strict confidentiality of personal information assured. This research does not cause harm to the human body and does not involve sensitive personal information or commercial interests. This study complies with the principles expressed in the Declaration of Helsinki. Moreover, this research complies with the ethical exemption requirements of the Ethical Review Measures for Life Sciences and Medical Research Involving Humans promulgated by China, and can be exempted from ethical review.

## 3. Results

### Demographic characteristics

A total of 593 questionnaires were distributed in this study, achieving a response rate of 90%. Among the collected questionnaires (n = 534), 518 were valid, resulting in a validity rate of 97%.

The results indicated that the majority of respondents were female, comprising 56.76% of the sample. In terms of age distribution, the largest proportion (47.30%) fell within the 28–37 years range. Most participants were married, accounting for 77.22%. The predominant monthly salary bracket was between 4001 and 6000 yuan, representing 44.21% of respondents. The highest level of education for most participants was either junior college or an undergraduate degree, at 87.64%. A significant number of the physicians did not hold professional titles, constituting 39.00%. The respondents had 0–5 years of work experience, accounting for 33.40%. Over the past three years, a significant majority of the physicians have participated in antimicrobial agents knowledge training, accounting for 89.96% of the total. Among these, 75.05% attended 1–5 sessions, primarily organized by various levels of health departments (90.11%). The main sources of antimicrobial agents knowledge included clinical medication guidelines (92.28%), clinical work experience (82.43%), and guidance from superior hospitals (72.97%). Detailed demographic characteristics of the surveyed primary care institution physicians are presented in Table 1.

**Table 1:** Demographic characteristics of physicians.

Characteristics	Category	No.(%)
Sex	Male	224(43.24)
	Female	294(56.76)
Age group	18-27 years	81(15.64)
	28-37 years	245(47.30)
	38-47 years	126(24.32)
	≥48 years	66(12.74)
Marital status	Unmarried	104(20.08)
	Married	400(77.22)
	Divorced	12(2.32)
	Widowed	2(0.38)
Work duration	0-5 years	173(33.40)
	6-10 years	129(24.90)
	11-15 years	79(15.25)
	16-20 years	53(10.23)
	21-25 years	35(6.76)
	26-30 years	35(6.76)
	≥31 years	14(2.70)
Professional title	No title	202(39.00)
	Resident physician	167(32.24)
	Attending physician	124(23.94)
	Associate chief physician	23(4.44)
	Chief physician	2(0.39)
Education	Technical secondary/High school	55(10.62)
	Junior college/Undergraduate	454(87.64)
	Master’s degree or above	9(1.74)
Monthly salary	2000-4000 RMB	204(39.38)
	4001-6000 RMB	229(44.21)
	6001-8000 RMB	64(12.36)
	8001−12,000 RMB	16(3.09)
	More than 12,000 RMB	5(0.97)
Have you attended any training on the appropriate use of antimicrobial agents in the past 3 years	Yes	466(89.96)
	No	52(10.04)
Number of training sessions	1-5 times	350(75.05)
	6-10 times	83(17.85)
	≥11 times	33(7.10)
Which units organize the training	Health department	419(90.11)
	Chinese center for control and prevention	224(48.17)
	Other government organizations	56(12.04)
	Colleges	32(6.88)
	Other	76(16.13)
In addition to training, obtain sources of information about antimicrobial agents use	Guidelines for clinical use of antimicrobial agents	478(92.28)
	Work experience in clinical	427(82.43)
	Academic articles	187(36.10)
	Academic promotion	149(28.76)
	Books related to continuing medical education	354(68.34)
	TV, Internet, newspapers and other media	237(45.75)
	Guidance from superior hospitals	378(72.97)
	Discussion among colleagues	284(54.83)
	Others	1(0.19)

### The scores of each dimension of the questionnaire

This questionnaire is structured around three dimensions: prescription KAP on antimicrobial agents. Higher scores in each dimension reflect greater levels of knowledge about antimicrobial prescription, more appropriate attitudes towards prescribing antimicrobial agents, and better practices in prescribing antimicrobial agents among the study participants. Specifically, the average score for prescription knowledge of antimicrobial agents among the physicians was 3.98 ± 1.85 (on a scale from 1 to 10). The average prescription attitude score was 41.97 ± 4.59 (ranging from 21 to 55), and the average prescription practice score was 40.01 ± 5.63 (ranging from 18 to 50) (Table 2).

**Table 2:** The scores of each dimension.

Dimensions	Minimum	Maximum	(Mean ± S)
Prescription knowledge	1	10	3.98 ± 1.85
Prescription attitudes	21	55	41.97 ± 4.59
Prescription practices	18	50	40.01 ± 5.63

### The physicians’ prescription knowledge scores on antimicrobial agents

The prescription knowledge dimension survey revealed that the item with the lowest score was K9 (Mycoplasma pneumonia can choose what antimicrobial agents to treat?), averaging 0.08 ± 0.28 points. The highest-scoring item was K6 (The purpose of the antimicrobial prescription evaluation system), with an average score of 0.94 ± 0.24.

**Fig 2:**
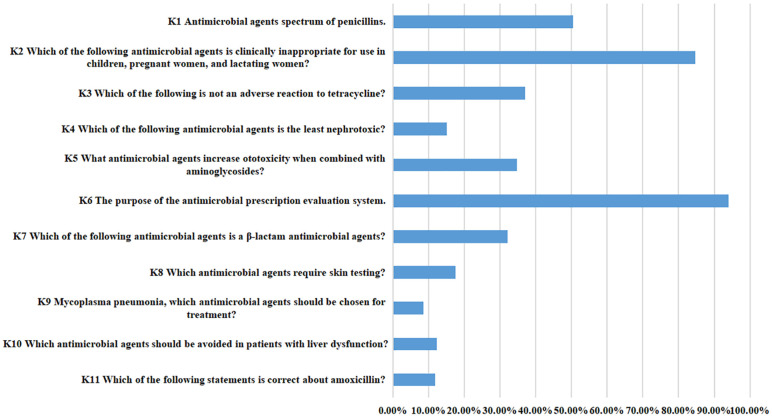
The ccorrect rate of physicians’ knowledge regarding antimicrobial prescriptions.

The physicians demonstrated satisfactory knowledge regarding the antimicrobial prescription evaluation system, with 94.02% correctly answering this question. Additionally, 84.75% of respondents accurately identified which antimicrobial agents are clinically inappropriate for use in children, pregnant women, and lactating women. However, only 8.49% knew the appropriate antimicrobial agents for treating Mycoplasma pneumoniae, only 11.78% had a relatively good understanding of amoxicillin, and only 12.36% correctly identified which antimicrobial agents should be avoided in patients with liver dysfunction (Table 3 and Fig 2).

**Table 3:** Responses to the questionnaire on antimicrobial prescription knowledge.

Prescription knowledge questions	(Mean ± S)	Correct rate(%)
**K1** Antimicrobial agents spectrum of penicillins.	0.50 ± 0.50	50.39%
**K2** Which of the following antimicrobial agents is clinically inappropriate for use in children, pregnant women, and lactating women?	0.85 ± 0.36	84.75%
**K3** Which of the following is not an adverse reaction to tetracycline?	0.37 ± 0.48	37.07%
**K4** Which of the following antimicrobial agents is the least nephrotoxic?	0.15 ± 0.36	15.06%
**K5** What antimicrobial agents increase ototoxicity when combined with aminoglycosides?	0.35 ± 0.48	34.75%
**K6** The purpose of the antimicrobial prescription evaluation system.	0.94 ± 0.24	94.02%
**K7** Which of the following antimicrobial agents is a β-lactam antimicrobial agents?	0.32 ± 0.47	32.05%
**K8** Which antimicrobial agents require skin testing?	0.18 ± 0.38	17.57%
**K9** Mycoplasma pneumonia, which antimicrobial agents should be chosen for treatment?	0.08 ± 0.28	8.49%
**K10** Which antimicrobial agents should be avoided in patients with liver dysfunction?	0.12 ± 0.33	12.36%
**K11** Which of the following statements is correct about amoxicillin?	0.12 ± 0.32	11.78%

### The physicians’ prescription attitude scores on antimicrobial agents

**Fig 3:**
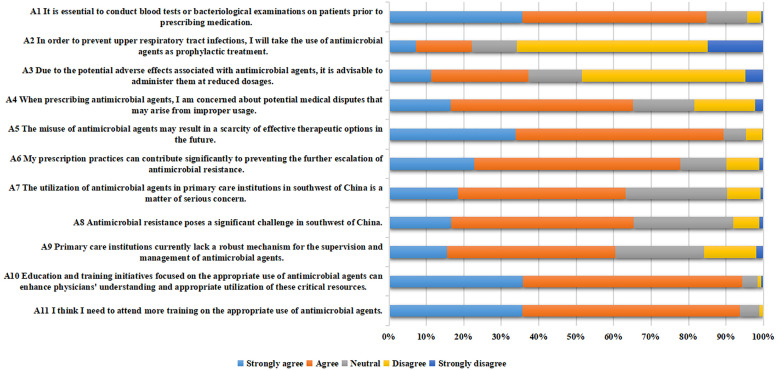
Physicians’ attitudes towards prescribing antimicrobial agents.

In the prescription attitude survey, the item with the lowest score was A3 (Because of the side effects of antimicrobial agents, they should be used in reduced doses), averaging 3.05 ± 1.16 points. The highest scoring items were A10 (Education and training on appropriate use of antimicrobial agents can improve physicians’ awareness of antimicrobial agents) and A11 (I think I need to attend more training on the appropriate use of antimicrobial agents), with average scores of 4.28 ± 0.64 and 4.28 ± 0.62, respectively. Some physicians believed that antimicrobial agents should be administered in reduced doses due to concerns about side effects (11.20% strongly agreed and 26.06% agreed). Regarding upper respiratory tract infections, a number of the physicians considered prescribing antimicrobial agents as a preventative medicine (7.14% strongly agreed and 15.06% agreed). The majority of the physicians believed that their prescription practices can help prevent the further escalation of antimicrobial resistance (22.78% strongly agreed and 55.02% agreed). More than half of the physicians considered the inappropriate use of antimicrobial agents in primary care institutions in southwest of China to be a serious concern (18.34% strongly agreed and 44.79% agreed), and they recognize the need for additional training to promote more appropriate use of antimicrobial agents (35.52% strongly agreed and 58.11% agreed) (Table 4 and Fig 3).

**Table 4:** Responses to the questionnaire on antimicrobial prescription attitude.

Prescription attitude questions	Strongly agree%	Agree%	Neutral%	Disagree%	Strongly disagree%	(Mean ± S)
**A1** It is essential to conduct blood tests or bacteriological examinations on patients prior to prescribing medication.	35.52%	49.23%	10.81%	3.86%	0.58%	4.15 ± 0.80
**A2** In order to prevent upper respiratory tract infections, I will take the use of antimicrobial agents as prophylactic treatment.	7.14%	15.06%	11.97%	50.97%	14.86%	3.51 ± 1.13
**A3** Due to the potential adverse effects associated with antimicrobial agents, it is advisable to administer them at reduced dosages.	11.20%	26.06%	14.29%	43.63%	4.83%	3.05 ± 1.16
**A4** When prescribing antimicrobial agents, I am concerned about potential medical disputes that may arise from improper usage.	16.41%	48.65%	16.41%	16.22%	2.32%	3.61 ± 1.02
**A5** The misuse of antimicrobial agents may result in a scarcity of effective therapeutic options in the future.	33.78%	55.60%	5.98%	4.25%	0.39%	4.18 ± 0.76
**A6** My prescription practices can contribute significantly to preventing the further escalation of antimicrobial resistance.	22.78%	55.02%	12.36%	8.69%	1.16%	3.90 ± 0.89
**A7** The utilization of antimicrobial agents in primary care institutions in southwest of China is a matter of serious concern.	18.34%	44.79%	27.22%	8.88%	0.77%	3.71 ± 0.89
**A8** Antimicrobial resistance poses a significant challenge in southwest of China.	16.60%	48.65%	26.64%	6.95%	1.16%	3.73 ± 0.86
**A9** Primary care institutions currently lack a robust mechanism for the supervision and management of antimicrobial agents.	15.44%	44.98%	23.75%	13.90%	1.93%	3.58 ± 0.97
**A10** Education and training initiatives focused on the appropriate use of antimicrobial agents can enhance physicians’ understanding and appropriate utilization of these critical resources.	35.71%	58.69%	4.05%	0.97%	0.58%	4.28 ± 0.64
**A11** I think I need to attend more training on the appropriate use of antimicrobial agents.	35.52%	58.11%	5.21%	0.97%	0.19%	4.28 ± 0.62

### The physicians’ prescription practice scores on antimicrobial agents

In the prescription practice survey, item P2 (For general illness, I prefer to prescribe broad-spectrum antimicrobial agents,over narrow-spectrum antimicrobial agents) received the lowest score with an average of 3.47 ± 1.17 points, while item P9 (The proportion of the number of prescriptions I filled in the past week that contained antimicrobials was approximately) achieved the highest average score of 4.50 ± 0.78.

**Fig 4:**
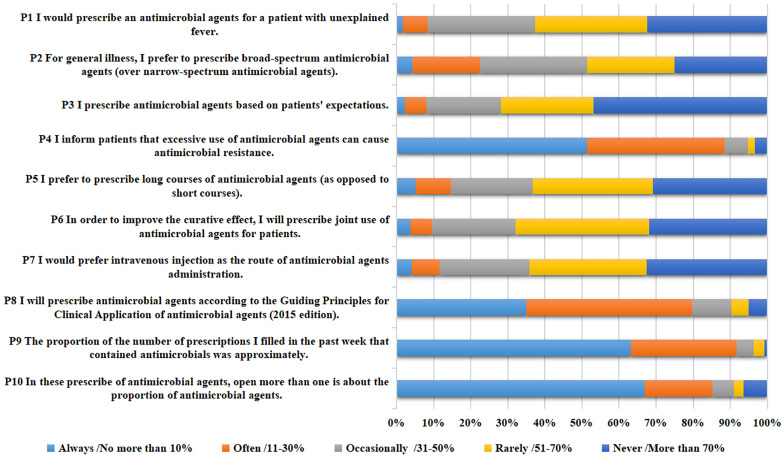
The responses regarding physicians’ antimicrobial prescription practice.

When reflecting on their experiences in prescribing antimicrobial agents, some physicians exhibited a tendency to prescribe broad-spectrum antimicrobial agents for common diseases (4.25% always and 18.34% often). However, it is reassuring that the most physicians inform patients about the risks of excessive antimicrobial agents use leading to resistance (51.35% always and 37.07% often). Additionally, the physicians referred to the “Guiding Principles for the Clinical Application of Antimicrobial Agents (2015 Edition)” when prescribing antimicrobial agents (34.94% always and 44.79% often). 63.13% of the physicians reported that the proportion of prescriptions containing antimicrobial agents over the past week was less than 10%. 66.99% of participants prescribed more than one antimicrobial agent in approximately 10% or fewer cases (Table 5 and Fig 4).

**Table 5:** Responses to the questionnaire on antimicrobial prescription practice.

Prescription practice questions	Always%	Often%	Occasionally %	Rarely%	Never%	(Mean ± S)
**P1** I would prescribe an antimicrobial agents for a patient with unexplained fever.	1.74%	6.56%	29.15%	30.12%	32.43%	3.85 ± 1.01
**P2** For general illness, I prefer to prescribe broad-spectrum antimicrobial agents (over narrow-spectrum antimicrobial agents).	4.25%	18.34%	28.76%	23.55%	25.10%	3.47 ± 1.17
**P3** I prescribe antimicrobial agents based on patients’ expectations.	2.12%	5.79%	20.27%	24.90%	46.91%	4.09 ± 1.04
**P4** I inform patients that excessive use of antimicrobial agents can cause antimicrobial resistance.	51.35%	37.07%	6.37%	1.93%	3.28%	4.31 ± 0.92
**P5** I prefer to prescribe long courses of antimicrobial agents (as opposed to short courses).	5.02%	9.65%	22.01%	32.43%	30.89%	3.75 ± 1.14
**P6** In order to improve the curative effect, I will prescribe joint use of antimicrobial agents for patients.	3.67%	5.79%	22.59%	36.10%	31.85%	3.87 ± 1.04
**P7** I would prefer intravenous injection as the route of antimicrobial agents administration.	4.05%	7.53%	24.32%	31.47%	32.63%	3.81 ± 1.10
**P8** I will prescribe antimicrobial agents according to the Guiding Principles for Clinical Application of antimicrobial agents (2015 edition).	34.94%	44.79%	10.62%	4.63%	5.02%	4.00 ± 1.05
Proportion of prescriptions prescribed						
Category	No more than 10%	11-30%	31-50%	51-70%	More than 70%	
**P9** The proportion of the number of prescriptions I filled in the past week that contained antimicrobials was approximately.	63.13%	28.57%	4.63%	2.90%	0.77%	4.50 ± 0.78
**P10** In these prescribe of antimicrobial agents, open more than one is about the proportion of antimicrobial agents.	66.99%	18.15%	5.79%	2.70%	6.37%	4.37 ± 1.13

### Uni-variate analysis

In the field of medicine and health, the results of statistical analysis are often presented in table form, which helps to present the data more clearly and concisely [21–22]. The results indicated that the uni-variate analysis of the total antimicrobial prescription knowledge and attitudes score in relation to the demographic characteristics of physicians was not statistically significant. Additionally, the overall score for antimicrobial prescription practices among the physicians was significantly correlated with work duration, age, monthly income, and professional title (*P* < 0.05) (Table 6).

**Table 6:** Association of demographic characteristics and dimension total scores based on uni-variate analysis.

Variables	No. (%)	Prescription knowledge			Prescription attitude			Prescription practice		
		Prescription knowledge scores (Mean ± S)	*t/F*	*P*	Prescription attitude scores(Mean ± S)	*t/F*	*P*	Prescription practice scores(Mean ± S)	*t/F*	*P*
Sex										
Male	294 (56.76)	3.93 ± 1.81	−0.811	0.417	41.68 ± 4.67	−1.686	0.092	40.39 ± 5.37	1.760	0.079
Female	224 (43.24)	4.06 ± 1.89			42.36 ± 4.46			39.51 ± 5.94		
Age group										
18-27 years	81 (15.64)	4.17 ± 1.69	1.009	0.388	41.72 ± 4.44	0.727	0.536	40.51 ± 5.09	2.835	**0.038**
28-37 years	245 (47.30)	4.06 ± 1.87			42.07 ± 4.57			39.32 ± 5.70		
38-47 years	126 (24.32)	3.83 ± 1.85			41.63 ± 4.62			40.33 ± 5.67		
≥48 years	66 (12.74)	3.77 ± 1.91			42.58 ± 4.79			41.35 ± 5.71		
Marital status										
Unmarried	104 (20.08)	4.33 ± 1.79	2.194	0.088	42.12 ± 4.58	2.084	0.101	40.26 ± 5.27	0.568	0.636
Married	400 (77.22)	3.92 ± 1.86			41.97 ± 4.59			39.99 ± 5.65		
Divorced	12 (2.32)	3.42 ± 1.51			42.33 ± 3.94			39.25 ± 8.08		
Widowed	2 (0.38)	2.50 ± 2.12			34.00 ± 1.41			35.50 ± 6.36		
Work duration										
0-5 years	173(33.40)	3.90 ± 1.865	1.054	0.390	41.34 ± 4.923	2.089	0.061	38.41 ± 6.080	4.799	**<0.001**
6-10 years	129(24.90)	4.05 ± 1.980			42.50 ± 4.776			40.21 ± 5.515		
11-15 years	79(15.25)	3.87 ± 1.712			42.24 ± 3.450			40.33 ± 5.380		
16-20 years	53(10.23)	4.49 ± 1.867			41.21 ± 3.997			41.55 ± 4.313		
21-25 years	35(6.76)	3.69 ± 1.843			41.89 ± 5.279			41.03 ± 5.649		
26-30 years	35(6.76)	4.09 ± 1.652			43.77 ± 4.291			41.91 ± 4.068		
≥31 years	14(2.70)	3.64 ± 1.277			42.07 ± 4.009			43.07 ± 5.636		
Professional title										
No title	202 (39.00)	4.03 ± 1.85	0.141	0.967	41.72 ± 4.45	0.562	0.691	40.41 ± 4.90	2.522	**0.040**
Resident physician	167 (32.24)	3.96 ± 1.78			42.28 ± 4.60			39.14 ± 5.98		
Attending physician	124 (23.94)	3.98 ± 1.92			42.06 ± 4.94			40.50 ± 6.02		
Associate chief physician	23 (4.44)	3.78 ± 2.02			41.30 ± 3.83			40.87 ± 6.31		
Chief physician	2 (0.39)	3.50 ± 0.71			44.00 ± 4.24			32.50 ± 2.12		
Education										
Technical secondary /High school	55 (10.62)	3.84 ± 1.87	1.008	0.366	41.91 ± 5.15	2.413	0.091	39.95 ± 5.51	0.004	0.996
Junior college/ Undergraduate	454 (87.64)	4.02 ± 1.85			42.05 ± 4.47			40.02 ± 5.64		
Master’s degree or above	9 (1.74)	3.22 ± 1.39			38.67 ± 5.96			40.00 ± 6.80		
Monthly salary										
2000-4000 RMB	204 (39.38)	3.88 ± 1.83	0.596	0.666	42.15 ± 4.45	1.058	0.377	40.19 ± 5.40	2.648	**0.033**
4001-6000 RMB	229 (44.21)	4.08 ± 1.89			41.70 ± 4.73			39.48 ± 5.85		
6001-8000 RMB	64 (12.36)	3.95 ± 1.75			42.67 ± 4.14			41.34 ± 5.26		
8001−12,000 RMB	16 (3.09)	4.19 ± 1.60			41.69 ± 5.67			41.44 ± 5.20		
More than 12,000 RMB	5 (0.97)	3.20 ± 2.49			39.40 ± 5.13			35.20 ± 7.16		
Have you attended any training on the appropriate use of antimicrobial agents in the past 3 years										
Yes	465 (89.96)	3.98 ± 1.86	0.151	0.880	41.91 ± 4.50	0.814	0.419	40.11 ± 5.77	−1.528	0.131
No	52 (10.04)	4.02 ± 1.76			42.54 ± 5.36			39.13 ± 4.18		

### Multivariate analysis

Multiple linear regression analysis was employed to conduct multi-variate analysis. Variables with a *P* < 0.2 from the uni-variate analysis were included as independent variables in the multi-variate model. The dependent variable comprised the total scores of each dimension: antimicrobial prescription KAP. The assignment of independent variables is detailed in S3 Appendix.

The results of the multi-variate analysis indicated that the number of marital status was a significant determinant of the physicians’ antimicrobial prescription knowledge and attitudes (*P* < 0.05). Specifically, physicians who have experienced the loss of a spouse have significantly lower knowledge of antimicrobial prescription compared to those without marital experience. Physicians who have experienced divorce have a significantly lower level of attitude towards the prescription of antimicrobial agents than those who without marital experience.

The work duration were identified as key factors influencing prescription attitudes and practices toward antimicrobial prescriptions (*P* < 0.05). Physicians with 6–10 years and 26–30 years of working experience scored significantly higher in terms of the appropriate attitude towards prescribing antimicrobial agents than those with 0–5 years of working experience. In terms of the practice of prescribing antimicrobial agents, physicians with more than five years of working experience scored significantly higher than those with zero to five years of working experience (Table 7).

**Table 7:** Multi-variable linear regression analyses on factors associated with dimension total scores.

Variables		*β*	*Se*	*Beta*	*t*	*P*
Prescription knowledge dimension						
Marital status						
	Unmarried (reference)					
	Married	−0.910	0.561	−0.074	−1.623	0.105
	Divorced	−1.827	1.313	−0.061	−1.392	0.165
	Widowed	−0.409	0.202	−0.093	−2.022	**0.044**
Prescription attitude dimension						
Marital status						
	Unmarried (reference)					
	Married	0.824	1.404	0.027	0.587	0.558
	Divorced	−6.768	3.328	−0.092	−2.033	**0.043**
	Widowed	−0.063	0.513	−0.006	−0.124	0.902
Work duration						
	0-5 years (reference)					
	6-10 years	1.160	0.528	0.109	2.198	**0.028**
	11-15 years	0.804	0.617	0.063	1.303	0.193
	16-20 years	−0.225	0.720	−0.015	−0.312	0.755
	21-25 years	0.623	0.843	0.034	0.739	0.460
	26-30 years	2.517	0.850	0.138	2.960	**0.003**
	≥31 years	1.048	1.280	0.037	0.818	0.414
Education						
	High school/technical secondary school (reference)					
	Junior college/undergraduate	0.188	0.666	0.013	0.282	0.778
	Master’s degree or above	−2.476	1.705	−0.071	−1.453	0.147
Sex		0.798	0.409	0.086	1.952	0.052
Prescription practice dimension						
Age group						
	18-27 years (reference)					
	28-37 years	−0.791	0.742	−0.070	−1.066	0.287
	38-47 years	−0.263	0.825	−0.020	−0.318	0.750
	≥48 years	0.957	0.946	0.057	1.011	0.312
Work duration						
	0-5 years(reference)					
	6-10 years	1.682	0.643	0.129	2.614	**0.009**
	11-15 years	1.851	0.748	0.118	2.475	**0.014**
	16-20 years	2.821	0.867	0.152	3.254	**0.001**
	21-25 years	2.359	1.025	0.105	2.301	**0.022**
	26-30 years	3.504	1.028	0.156	3.409	**0.001**
	≥31 years	4.659	1.547	0.134	3.011	**0.003**
Monthly salary						
	2000-4000 RMB (reference)					
	4001-6000 RMB	−0.590	0.588	−0.052	−1.004	0.316
	6001-8000 RMB	1.095	0.873	0.064	1.254	0.210
	8001−12,000 RMB	0.460	1.518	0.014	0.303	0.762
	more than 12,000 RMB	−3.353	2.892	−0.058	−1.160	0.247
Training experience		0.527	0.820	0.028	0.643	0.521
Sex		−0.863	0.496	−0.076	−1.740	0.083
Professional title						
	No title (reference)					
	Resident physician	−1.038	0.639	−0.086	−1.623	0.105
	Attending physician	0.188	0.749	0.014	0.251	0.802
	Associate chief physician	0.051	1.422	0.002	0.036	0.972
	Chief physician	−5.442	4.149	−0.060	−1.312	0.190

## 4. Discussion

This study conducted a questionnaire survey on the KAP of 518 physicians in the southwest region of China regarding the antimicrobial prescription, aiming to identify the weak links and related influencing factors in the KAP of physicians in the prescription of antimicrobial agents. The research results provide important references for the future formulation and optimization of antimicrobial stewardship policies, as well as the education and training programs for physicians. In the framework of the KAP theory, there exists a dynamic circular relationship among KAP. Knowledge serves as the foundation for the formation of attitude and practice. Attitude, as a mediating variable, influences the occurrence of practice, while practice is manifested as specific practical outcomes. Meanwhile, problems exposed during the practical process can also have a reverse effect on the knowledge and attitudinal levels, promoting the continuous improvement of an individual’s KAP level [23–24]. The relevant theoretical framework also indirectly supports this statement. Self-Efficacy Theory indicates that knowledge is one of the foundations for the formation of self-efficacy. By mastering relevant knowledge and skills, an individual’s experience of successfully completing tasks will enhance their self-efficacy, fostering the belief that “I can do it”, and thereby encouraging them to take corresponding actions. Expectancy-Value Theory indicates that knowledge can influence an individual’s expectations and value judgments regarding a task, thereby shaping corresponding beliefs and ultimately affecting practice [25–26].

In the prescription knowledge dimension, it consisted of 11 questions. Among the physicians’ responses to questions regarding antimicrobial prescriptions, only 3 questions demonstrated a correct response rate exceeding 50%. The results indicated that physicians in southwest China had a low overall score regarding antimicrobial prescription knowledge. This finding aligns with multiple studies from Germany, the United Kingdom, and the United States, which similarly indicate a relatively insufficient level of antimicrobial prescription-related knowledge among primary care institutions physicians [27–30]. Most physicians in primary care institutions in southwestern China hold junior college or lower level of education. The relatively low overall educational attainment and professional competence of these physicians, a lack of relevant education and training, contribute to their insufficient understanding of antimicrobial use [31–32].

This study revealed that approximately 91.51% of physicians were unaware of which antimicrobial agents could be used for the treatment of mycoplasma pneumonia, indicating a significant gap in their knowledge regarding varieties of use for antimicrobial agents. The rate of updating antimicrobial prescription-related knowledge among physicians in primary care institutions is relatively slow. If they fail to keep abreast of the latest guidelines, they may inadvertently prescribe inappropriate medications when faced with specific indications [33–34]. Additionally, a mere 11.78% of physicians provided correct answers to questions regarding amoxicillin. Despite its excellent antimicrobial efficacy, which has made it a preferred choice for many physicians and patients, the low level of understanding about amoxicillin among these physicians increases the risk of its misuse [35–36]. Therefore, enhancing the awareness and attention of the physicians regarding varieties of antimicrobial agents use and common medications is crucial. In clinical practice, drugs such as erythromycin and rifamycin should be avoided in patients with impaired liver function. However, only 12.36% of the physicians in this study provided correct answers to related questions. The inappropriate use of antimicrobial agents can cause liver injury lasting 1–3 weeks, in more severe cases, it may result in acute liver failure or autoimmune reactions. Therefore, physicians must pay close attention to contraindications and exercise caution when prescribing antimicrobial agents [37]. Furthermore, in clinical settings, the concurrent use of aminoglycoside antimicrobial agents and furosemide can lead to ototoxicity, damaging the cochlea and vestibular system, and causing symptoms such as tinnitus, hearing loss, or even deafness [38]. Thus, these two drugs should not be prescribed together. This study revealed that only 34.75% of the physicians had a clear understanding of this issue. These physicians demonstrated insufficient prescription knowledge regarding antimicrobial agents, particularly in selecting appropriate antimicrobial agents for specific indications, and recognizing contraindications and precautions. These areas should be prioritized by relevant departments and institutions for targeted education, training, and intervention in the future. Therefore, it is necessary to provide education and training on the appropriate use of antimicrobial agents to physicians. Hospital management departments can collaborate with relevant administrative departments and research institutions to develop educational materials that can fill the cognitive gaps of physicians [39–40]. On the other hand, relevant research shows that currently in China, antimicrobial stewardship programs such as prescription review, educational intervention, and feedback intervention have been carried out, significantly reducing the inappropriate use of antimicrobial agents [41–43]. Subsequent research [42] by our team has shown that, considering the actual situation of primary care institutions, the three intervention measures of prescription review, education and training, and feedback intervention can be combined to enhance physicians’ awareness of the appropriate use of antimicrobial agents.

In the prescription attitude dimension, this study revealed that physicians’ antimicrobial prescription attitudes overall scored are average, aligning with findings from studies in Poland and the United States. These studies suggests an average level of rationality in the attitudes of these physicians toward antimicrobial prescription [44–45].

In the prescriptions practice dimension, this study revealed that physicians in southwest China achieved a high overall score in the antimicrobial prescription practice dimension. Consistent with the research findings from Japan and Poland, these studies highlights that physicians demonstrate a relatively high level of competence in antimicrobial prescription practices [44,46].

The results of the multivariate analysis indicated that the working years of physicians is a significant influencing factor on their attitudes and practices towards prescribing antimicrobial agents (P < 0.05). Among them, the attitude score of physicians with 0–5 years of working experience is significantly lower than that of physicians with 6–10 and 26–30 years of working experience. The practice score of physicians with 0–5 years of working experience is lower than that of physicians with more than 5 years of working experience. This suggests that working years have a positive impact on physicians’ attitudes and practices towards prescribing antimicrobial agents, which is consistent with the research results of El-Sokkary R et al [47]. The reason is that as the working years and experience of physicians increase, their mastery of drug dosage, interactions and indications can be significantly improved, thereby reducing prescription errors [48]. For physicians with shorter working experience, authoritative physicians with many years of experience can be invited to provide education and training on the appropriate use of antimicrobial agents and the mechanism of antimicrobial resistance to young physicians. Clinical scenario simulation can also be used to gradually change the prescription practice and habits of physicians. Then, the dynamic changes in the KAP of young physicians in antimicrobial prescriptions can be judged through the annual prescription review results [49–50].

### Limitations

There are several limitations to this study. Firstly, selection bias may have occurred as the physicians who received the questionnaire link might have declined to participate due to personal preferences or time constraints. Secondly, the reliance on self-report questionnaires could introduce response bias, where respondents may provide answers that align with socially desirable norms rather than reflecting their genuine beliefs or opinions. Thirdly, the study was geographically restricted to southwest China, which may constrain its generalizability. Future research should investigate prescription KAP in other regions of China to gain a comprehensive understanding of the national prescription KAP landscape. Despite these limitations, the findings of this study offer valuable insights into the KAP of antimicrobial prescriptions among the physicians. Furthermore, they provide some directions for the design, enhancement, and implementation of future policies, educational programs, training initiatives, and interventions.

### Conclusion

The results of this study revealed that the knowledge level of antimicrobial prescriptions among physicians in primary care institutions in southwest China was relatively low, while their prescription attitudes and practices were generally favorable but still require improvement. From the standpoint of influencing factors, the gender and years of experience of physicians significantly impacted their levels of KAP concerning antimicrobial use. The findings of this study can provide valuable insights for the relevant management departments and research institutions regarding physicians in formulating educational and training materials on contraindications, precautions, and indications of antimicrobial agents, and others, guiding the optimization of regulatory policies for the appropriate use of antimicrobial agents in primary care institutions. Further research is still needed in the future to explore which other social and external factors will affect the antimicrobial agents awareness level of physicians.

## Supporting information

S1 Appendix The 2015 edition of China’s basic principles for the clinical application of antimicrobial agents. (DOCX)

S2 Appendix The aggregated physician data. (XLSX)

S3 Appendix Fundamental variable assignment for the physicians. (DOCX)

S4 Appendix Questionnaire in Chinese. (DOCX)

S5 Appendix Questionnaire in English. (DOCX)

S6 Appendix Questionnaire scoring table. (XLSX)
