# Supplementary material for: Antimicrobial prescription KAP among physicians in primary care institutions in Southwest China
Source: PLoS One. 2025 Nov 13;20(11):e0335484. doi: 10.1371/journal.pone.0335484 (PMC12614571; doi:10.1371/journal.pone.0335484)
Supplement: S3 Appendix — (DOCX) [file pone.0335484.s003.docx]

Fundamental variable assignment for the physicians

| Variables | Name of variable | Assignment |
| --- | --- | --- |
| Age group | X1 | 1=18-27 years, 2=28-37 years, 3=38-47 years,  4= ≥48 years |
| Marital status | X2 | 1= unmarried, 2= married, 3= divorced,  4= widowed |
| Work duration | X3 | 1=0-5 years, 2=6-10 years, 3=11-15 years,  4=16-20 years，5=21-25 years，6=26-30 years， 7= ≥31 years |
| Monthly salary | X4 | 1=200-4000 RMB,  2=4001-6000 RMB,  3=6001-8000 RMB,  4= 8001-12,000 RMB,  5= more than 12,000 RMB |
| Training experience | X5 | 1= no, 2= yes |
| Sex | X6 | 1= female, 2= male |
| Education | X7 | 1= high school/technical secondary school,  2= junior college/undergraduate,  3= master's degree or above |
| Professional title | X8 | 1= no title, 2= resident physician, 3= attending physician, 4= associate chief physician,  5= chief physician |
