# Supplementary material for: Antimicrobial prescription KAP among physicians in primary care institutions in Southwest China
Source: PLoS One. 2025 Nov 13;20(11):e0335484. doi: 10.1371/journal.pone.0335484 (PMC12614571; doi:10.1371/journal.pone.0335484)
Supplement: S4 Appendix — (DOCX) [file pone.0335484.s004.docx]

**基层医疗机构门诊医生****抗菌药物认知、处方态度和实践评价问卷**

**第一部分：个人基本情况**

1. 您的性别：

①男 ②女

2. 您的年龄：__________岁。

3.您的婚姻状况：

①未婚 ②已婚 ③离婚 ④丧偶

4. 您从事医疗工作______年。

5. 您的职称：

①无职称 ②住院医师 ③主治医师 ④副主任医师 ⑤主任医师

6. 您的最高学历：

① 中专/高中 ②大专/本科 ③硕士及以上

7. 您的平均月工资收入（包括工资、奖金、津贴、福利等）：

①2000元～4000 元 ②4001~6000元 ③6001~8000元 ④8001-12000元⑤12000元以上

8. 在过去3年中，您是否参加过关于合理使用抗菌药物的培训（包括讲座、研讨会、医学继续教育课程等）？（）

①是 ②否（选否，则跳到第11题）

9. 在过去3年中，您参加培训的次数为_____次。

10. 您参加培训由哪些单位组织？（）（多选题）

①各级卫健部门 ②各级疾控部门 ③其它政府组织

④高校 ⑤其它（请注明）____________

11. 除了培训外，您还从下列哪些来源获得抗菌药物的使用信息？（）（多选题）

①抗菌药物临床用药指南 ②临床工作经验 ③学术文章 ④学术推广 ⑤医学继续教育相关书籍 ⑥电视、网络、报纸等媒体 ⑦上级医院指导

⑧同事间讨论 ⑨其它（请注明）_________

**第二部分：抗菌药物认知情况**

为了解您对抗菌药物知识的掌握情况，需要您在下列选项中选出您认为正确的选项，调查结果不会用于任何考核，您可以放心作答。

1.青霉素的抗菌谱是（）。

①革兰阳性菌 ②革兰阴性球菌 ③螺旋体 ④以上均是

2. 下列抗菌药物中，哪种药物临床上不宜用于儿童、孕妇和哺乳期妇女（）。

① 头孢菌素类 ②氟喹诺酮类 ③大环内酯类 ④青霉素类

3.下列哪一个不是四环素的不良反应？（）

①二重感染 ②灰婴综合征 ③肝脏毒性 ④影响骨、牙生长

4.下列抗菌药物中，肾毒性最小的药物是？（）

①头孢唑林 ②头孢他啶 ③头孢曲松 ④头孢匹罗

5.与氨基糖苷类药合用可增加耳毒性的药物是（）。

①呋塞米 ②氯霉素 ③四环素 ④青霉素G

6.抗菌药物处方点评制度的目的是（）。

①统计处方数量 ②监督、规范抗菌药物使用 ③考核医生工作量

④挑选优质处方 ⑤对医院进行绩效考核

7. 下列抗菌药物中，属于$\beta$-内酰胺类抗菌药物的是？（）（多选题）

①红霉素 ②克林霉素 ③青霉素 ④头孢匹罗 ⑤氯霉素

8.需做皮试的药品包括（）。（多选题）

①青霉素G注射液 ②头孢唑啉钠注射液 ③硫酸链霉素注射液

④盐酸普鲁卡因注射液 ⑤盐酸林可霉素注射液

9.支原体肺炎可选用的抗菌药物有（）。（多选题）

① 四环素 ②氨基糖苷类 ③红霉素 ④青霉素 ⑤头孢唑林

10.临床上患者肝功能减退时避免应用的药物有哪些？（）（多选题）

① 青霉素类 ②头孢菌素类 ③红霉素 ④利福平 ⑤喹诺酮类

11. 下列表述中，对阿莫西林描述正确的是？（）（多选题）

①抗菌谱、抗菌活性与氨苄西林相似

②对肺炎的治疗较氨苄西林弱

③胃肠道吸收良好

④用于下呼吸道感染的治疗

⑤为对位羟基氨苄西林

**第三部分：抗菌药物处方态度情况**

1.我认为在开具处方前，需要对患者做血液检查或细菌学检验（）。

①非常同意 ②同意 ③ 中立 ④不同意 ⑤非常不同意

2.针对上呼吸道感染，我会开具抗菌药物作为预防性用药（）。

①非常同意 ②同意 ③ 中立 ④不同意 ⑤非常不同意

3. 因为抗菌药物具有副作用，所以要减量使用（）。

①非常同意 ②同意 ③ 中立 ④不同意 ⑤非常不同意

4.我在开具抗菌药物处方时，会担心因用药不当而引发医疗纠纷？（）
①非常同意 ②同意 ③中立 ④不同意 ⑤非常不同意

5.滥用抗菌药物会导致以后无药可用（）。

①非常同意 ②同意 ③中立 ④不同意 ⑤非常不同意

6.我的处方行为能预防抗菌药物耐药性的进一步升级（）。

①非常同意 ②同意 ③中立 ④不同意 ⑤非常不同意

7.贵州省基层医疗机构的抗菌药物不合理应用情况严峻（）。
①非常同意 ②同意 ③中立 ④不同意 ⑤非常不同意

8.贵州省的抗菌药物耐药性是一个严重的问题（）。

①非常同意 ②同意 ③中立 ④不同意 ⑤非常不同意

9.基层医院缺乏有效的抗菌药物监督管理机制（）。

①非常同意 ②同意 ③中立 ④不同意 ⑤非常不同意

10.抗菌药物合理用药教育培训能提升医生的抗菌药物认知水平（）。

①非常同意 ②同意 ③ 中立 ④不同意 ⑤非常不同意

11.我认为自己需要参加更多抗菌药物合理使用的培训（）。

①非常同意 ②同意 ③ 中立 ④不同意 ⑤非常不同意

**第四部分：抗菌药物处方实践情况**

1. 我会为不明原因发热患者开具抗菌药物（）。

# ①总是 ②经常 ③偶尔 ④极少 ⑤从不

1. 对于一般疾病，我更倾向于开具广谱抗菌药物（相较于窄谱抗菌药物）（）。

# ①总是 ②经常 ③偶尔 ④极少 ⑤从不

1. 我会因为患者期望而开具抗菌药物（）。

# ①总是 ②经常 ③偶尔 ④极少 ⑤从不

4. 我会告知患者过量服用抗菌药物会引起抗菌药物耐药性（）。

# ①总是 ②经常 ③偶尔 ④极少 ⑤从不

5.我更倾向于开具长疗程的抗菌药物（相较于短疗程）（）。

# ①总是 ②经常 ③偶尔 ④极少 ⑤从不

6.为了提高疗效，我会为患者开具联合使用抗菌药物的处方（）。

# ①总是 ②经常 ③偶尔 ④极少 ⑤从不

7.我会首选静脉注射作为抗菌药物的给药途径（）。

# ①总是 ②经常 ③偶尔 ④极少 ⑤从不

8.我会参考《抗菌药物临床应用指导原则（2015年版）》开具抗菌药物处方（）。

# ①总是 ②经常 ③偶尔 ④极少 ⑤从不

9.在过去一周内，我开具的含有抗菌药物的处方占总处方的数量比例约为（）。

①不高于10% ②11～30% ③31～50% ④51～70% ⑤70%以上

10.在这些开具抗菌药物的处方中，开具一种以上抗菌药物的比例约为（）。

①不高于10% ②11～30% ③31～50% ④51～70% ⑤70%以上
