# Supplementary material for: Antimicrobial prescription KAP among physicians in primary care institutions in Southwest China
Source: PLoS One. 2025 Nov 13;20(11):e0335484. doi: 10.1371/journal.pone.0335484 (PMC12614571; doi:10.1371/journal.pone.0335484)
Supplement: S5 Appendix — (DOCX) [file pone.0335484.s005.docx]

**Questionnaire for assessing physicians' knowledge, attitudes, and practices regarding antimicrobial** **prescriptions in primary care institutions**

**Part 1: Personal basic information**

1. Your sex:

① Male ② Female

2. Your age: __________.

3. Your marital status:

① Unmarried ② Married ③ Divorced ④ Widowed

4. You have been engaged in medical work for ______ years.

5. Your professional title:

① No professional title ② Resident physician ③ Attending physician

④ Associate chief physician ⑤ Chief physician

6. Your highest degree:

① Technical secondary /High school ② Junior college/Undergraduate

③ Master's degree or above

7. Your average monthly salary (including salary, bonus, allowance, welfare, etc.) :

① 2000-4000 RMB ② 4001-6000 RMB ③ 6001-8000 RMB

④ 8001-12,000 RMB ⑤ More than 12,000 RMB

8. In the past 3 years, have you participated in any training (including lectures, seminars, continuing medical education courses, etc.) on the appropriate use of antimicrobial agents? ( )

① Yes ② No (Choose no, then skip to question 11)

9. In the past 3 years, the number of times you have attended training is _____.

10. Which organization organized your training? ( ) (Multiple-choice question)

① Health departments ② Chinese center for control and prevention

③ Other government organizations ④ Colleges

⑤ Other (please specify) ____________

11. In addition to training, from which of the following sources do you obtain information on antimicrobial use? ( ) (Multiple-choice question)

① Guidelines for clinical use of antimicrobial agents

② Work experience in clinical

③ Academic articles ④ Academic promotion

⑤ Books related to continuing medical education

⑥ TV, Internet, newspapers and other media

⑦ Guidance from superior hospitals

⑧ Discussion among colleagues ⑨ Others (please specify) _________

**Part II: Prescription knowledge of antimicrobial agents**

To understand your grasp of the knowledge of antimicrobial agents, need you in the following options are selected option you think is right, the results of the survey will not use it for any evaluation, you can rest assured.

**K1** Antimicrobial agents spectrum of penicillins ( ).

1. Gram-positive bacteria ② Gram-negative cocci ③ Spirochete

④ All of the above are

**K2** Which of the following antimicrobial agents is clinically inappropriate for use in children, pregnant women, and lactating women? ( )

① Cephalosporins ② Fluoroquinolones ③ Macrolides ④ Penicillins

**K3** Which of the following is not an adverse reaction to tetracycline? ( )

1. Double infection ② Grey baby syndrome ③ Liver toxicity

④ Influence the growth of bone and teeth

**K4** Which of the following antimicrobial agents is the least nephrotoxic? ( )

① Cefazolin ② Ceftazidime ③ Ceftriaxone ④ Cefpirome

**K5** What antimicrobial agents increase ototoxicity when combined with aminoglycosides? ( )

① Furosemide ② Chloramphenicol ③ Tetracycline ④ Penicillin G

**K6** The purpose of the antimicrobial prescription evaluation system( ).

To count the number of prescriptions

To supervise and standardize the use of antimicrobial agents

③ To assess the workload of physicians

④ Selecting high quality prescriptions and

⑤ Conducting hospital performance appraisal

**K7** Which of the following antimicrobial agents is a β-lactam antimicrobial agents?（ ）(Multiple-choice question)

① Erythromycin ② Clindamycin ③ Penicillin ④ Cefpirol ⑤ Chloramphenicol

**K8** Which antimicrobial agents require skin testing?（ ）(Multiple-choice question)

Penicillin G injection ② Cefazolin sodium injection

③ Streptomycin sulfate injection

④ Procaine hydrochloride injection ⑤ Lincomycin hydrochloride injection

**K9** Mycoplasma pneumonia, which antimicrobial agents should be chosen for treatment? （ ）(Multiple-choice question)

① Tetracycline ② Aminoglycosides ③ Erythromycin ④ Penicillin ⑤ Cefazolin

**K10** Which antimicrobial agents should be avoided in patients with liver dysfunction? ( ) (Multiple-choice question)

① Penicillins ② Cephalosporins ③ Erythromycin ④ Rifampicin ⑤ Quinolones

**K11** Which of the following statements is correct about amoxicillin?( ) (Multiple-choice question)

① Antibacterial spectrum and antibacterial activity were similar to those of ampicillin

② The treatment of pneumonia is weaker than that of ampicillin

③ Good absorption in gastrointestinal tract

④ It is used in the treatment of lower respiratory tract infection

⑤ For para-hydroxyampicillin

**Part III: Prescription attitudes of antimicrobial agents**

**A1** It is essential to conduct blood tests or bacteriological examinations on patients prior to prescribing medication ( ).

① Strongly agree ② Agree ③ Neutral ④ Disagree ⑤ Strongly disagree

**A2** In order to prevent upper respiratory tract infections, I will take the use of antimicrobial agents as prophylactic treatment ( ).

1. Strongly agree ② Agree ③ Neutral ④ Disagree ⑤ Strongly disagree

**A3** Due to the potential adverse effects associated with antimicrobial agents, it is advisable to administer them at reduced dosages ( ).

1. Strongly agree ② Agree ③ Neutral ④ Disagree ⑤ Strongly disagree

**A4** When prescribing antimicrobial agents, I am concerned about potential medical disputes that may arise from improper usage ( ).

① Strongly agree ② Agree ③ Neutral ④ Disagree ⑤ Strongly disagree

**A5** The misuse of antimicrobial agents may result in a scarcity of effective therapeutic options in the future ( ).

① Strongly agree ② Agree ③ Neutral ④ Disagree ⑤ Strongly disagree

**A6** My prescription practices can contribute significantly to preventing the further escalation of antimicrobial resistance ( ).

1. Strongly agree ② Agree ③ Neutral ④ Disagree ⑤ Strongly disagree

**A7** The utilization of antimicrobial agents in primary care institutions in southwest of China is a matter of serious concern ( ).
① Strongly agree ② Agree ③ Neutral ④ Disagree ⑤ Strongly disagree

**A8** Antimicrobial resistance poses a significant challenge in southwest of China ( ).

① Strongly agree ② Agree ③ Neutral ④ Disagree ⑤ Strongly disagree

**A9** Primary care institutions currently lack a robust mechanism for the supervision and management of antimicrobial agents ( ).

① Strongly agree ② Agree ③ Neutral ④ Disagree ⑤ Strongly disagree

**A10** Education and training initiatives focused on the appropriate use of antimicrobial agents can enhance physicians' understanding and appropriate utilization of these critical resources ( ).

① Strongly agree ② Agree ③ Neutral ④ Disagree ⑤ Strongly disagree

**A11** I think I need to attend more training on the appropriate use of antimicrobial agents ( ).

① Strongly agree ② Agree ③ Neutral ④ Disagree ⑤ Strongly disagree

**Part 4: Prescription practices of antimicrobial agents**

**P1** I would prescribe an antimicrobial agents for a patient with unexplained fever ( ).

# ① Always ② Often ③ Occasionally ④ Rarely ⑤Never

**P2** For general illness, I prefer to prescribe broad-spectrum antimicrobial agents (over narrow-spectrum antimicrobial agents) ( ).

# ① Always ② Often ③ Occasionally ④ Rarely ⑤Never

**P3** I prescribe antimicrobial agents based on patients' expectations ( ).

# ① Always ② Often ③ Occasionally ④ Rarely ⑤Never

**P4** I inform patients that excessive use of antimicrobial agents can cause antimicrobial resistance ( ).

# ① Always ② Often ③ Occasionally ④ Rarely ⑤Never

**P5** I prefer to prescribe long courses of antimicrobial agents (as opposed to short courses) ( ).

# ① Always ② Often ③ Occasionally ④ Rarely ⑤Never

**P6** In order to improve the curative effect, I will prescribe joint use of antimicrobial agents for patients ( ).

# ① Always ② Often ③ Occasionally ④ Rarely ⑤Never

**P7** I would prefer intravenous injection as the route of antimicrobial agents administration ( ).

# ① Always ② Often ③ Occasionally ④ Rarely ⑤Never

**P8** I will prescribe antimicrobial agents according to the Guiding Principles for Clinical Application of antimicrobial agents (2015 edition) ( ).

# ① Always ② Often ③ Occasionally ④ Rarely ⑤Never

**P9** The proportion of the number of prescriptions I filled in the past week that contained antimicrobials was approximately ( ).

① No more than 10% ② 11-30% ③ 31-50% ④ 51-70% ⑤ More than 70%

**P10** In these prescribe of antimicrobial agents, open more than one is about the proportion of antimicrobial agents ( ).

① No more than 10% ② 11-30% ③ 31-50% ④ 51-70% ⑤ More than 70%
